# Evaluation of Co‐Production in Adolescent Health Research: A Mixed Methods Study From Born in Bradford

**DOI:** 10.1111/hex.70876

**Published:** 2026-10-15

**Authors:** David Ryan, Adam Whitehead, Amelia Hudak, Edie Lee, Isobel Dowling, Jennie Ellul, Jennifer Ingram, Katy A. Shire, Shahid Islam, John Wright, Rosemary R. C. McEachan

**Affiliations:** ^1^ Bradford Institute for Health Research, Bradford Teaching Hospitals NHS Foundation Trust Bradford Royal Infirmary Bradford UK; ^2^ School of Psychology University of Leeds Leeds UK; ^3^ School of Humanities and Social Sciences Leeds Beckett University Leeds UK

**Keywords:** born in Bradford, co‐production, evaluation, mixed methods, young people

## Abstract

**Background:**

Co‐production is often viewed as best practice for involving the public in research, while measures such as sharing power, reciprocity, and relationship‐building are seen as key guiding principles. However, implementing co‐production and its underlying principles remains challenging, with limited evaluative work examining how these principles are applied in practice, particularly within large‐scale studies and with young people. This study aimed to explore whether Born in Bradford's (BiB) co‐production values and principles have been embedded in BiB Age of Wonder (AoW), a longitudinal study following over 20,000 Bradford teenagers through secondary school and into early adulthood.

**Methods:**

This evaluation used a mixed‐methods approach. Participants included parents, young people, teachers, and community members. Quantitative data was collected via an online survey (*n* = 79). Qualitative data was collected via four focus groups (*n* = 17). The survey and focus group topic guide were co‐designed with young people, with questions designed to assess how well the principles and values of BiB's co‐production strategy had been embedded in AoW. Descriptive statistics were used to explore the survey responses. Qualitative data was analysed using framework analysis.

**Results:**

Survey and focus group findings indicated that co‐production group members mostly agreed or strongly agreed that the nine principles of co‐production had been embedded in AoW. However, ‘sharing power’ and ‘community engagement’ were found to be areas that could be improved, with participants calling for earlier involvement in research design, clearer feedback loops, and outreach beyond existing groups.

**Conclusions:**

We found evidence that the values and principles of co‐production have been embedded in AoW co‐production. Suggestions for improvement include shaping research agendas through earlier involvement, deeper engagement with schools and communities, and paid peer research posts. Future steps include (1) developing sustainable peer research strategies to deepen co‐production, and (2) incorporating perspectives from diverse stakeholders including budget holders, facilitators, researchers and public contributors in future evaluations.

**Patient or Public Contribution:**

Members of the public were involved in the study conceptualisation, methods development and implementation. The survey and focus group topic guide were co‐designed with young people from BiB Age of Wonder co‐production groups. Co‐production group members were also involved as participants in the surveys and focus groups.

## 1 Background

Co‐production is defined by the National Institute for Health and Care Research (NIHR) as an approach in which ‘researchers, practitioners and members of the public work together, sharing power and responsibility from the start to the end of the project, including the generation of knowledge’ [1]. Co‐production is often seen as best practice in terms of public involvement in research and is praised as an egalitarian approach to research [2, 3] that empowers community members and aligns research agendas with community priorities [4, 5]. Moreover, co‐production is thought to improve research quality, generate more applicable and relevant knowledge [6, 7], and improve translation of research evidence into practice and policy [6, 8, 9]. However, despite growing support, co‐production continues to struggle with a significant theory‐practice gap [10, 11]. Key contributors to this gap include a multitude of co‐production definitions [12], interchangeable terminology [2], a lack of transparent reporting of co‐production practices and challenges [13, 14], and structural barriers (e.g., funding timelines, academic traditions) [10, 15].

A promising development in the co‐production landscape has been a move away from proliferating definitions [12] to thinking of co‐production as operationalising key values and principles [12, 16, 17, 18, 19]. Previous research (including research by our group) has highlighted common principles of co‐production practice, including sharing power [1, 20, 21, 22], embracing reciprocity [1, 2, 23, 24], and building long term relationships [15, 19, 25]. It is thought that describing co‐production in this way may help bridge the theory‐practice gap [10, 16], by viewing co‐production “not as a linear approach but rather an ethos that shapes practice” [26]. That said, some have described principle‐based descriptions of co‐production as overly abstract [26], while previous research suggests that enacting all principles may not always be feasible, especially when research teams face resource constraints or are working with small co‐production groups [10]. These challenges highlight the need for further research and more transparent reporting on the methods and difficulties involved in applying co‐production principles and values across diverse contexts [16, 26, 27].

Another important gap in research and understanding is a lack of robust evaluations on co‐production [28]. One reason for this may be that co‐production is innately hard to evaluate. Co‐production necessitates a level of methodological flexibility and adaptability that can make it difficult to assign fixed outcomes (e.g., co‐production may prompt changes to study aims and protocols [29] or may involve extending or adapting study timelines to support group members' invovlement [16]). This may partly explain why systematic reviews have found much of the existing co‐production literature focuses on co‐production processes rather than describing what outcomes occurred because of co‐production [27, 30, 31]. Co‐production can also be difficult to evaluate due to it's greater emphasis placed on ‘softer’ and harder‐to‐measure outcomes such as trust, participant empowerment, and social cohesion compared to traditional research [16, 32, 33]. This is further compounded when we consider difficulties with establishing long‐term working with co‐production groups [27], challenges embedding and evaluating co‐production within resource and time limited projects, limited public involvement in existing co‐production evaluation design [28], and a lack of evaluative work with young people [16, 34].

### 1.1 Co‐Producing Born in Bradford's Age of Wonder

Born in Bradford (BiB) is a multi‐ethnic birth cohort in Bradford, UK which recruited 12,453 women with 13,776 pregnancies and 3448 of their partners between 2007 and 2011 [35, 36]. Co‐production and community engagement is central to BiB research [4, 37, 38, 39], and BiB's co‐production approach follows the principles and values of a bespoke co‐production strategy designed to provide a framework for engaging communities in all stages of the research process [37, 40]. This strategy was developed with researchers, decision‐makers and stakeholders through an iterative process with up to 100 community members. It describes meaningful co‐production across three core values (equality, agency, reciprocity) and outlines nine guiding principles to support researchers engaged in co‐production activities (see Supporting Information: Appendices [Link], [Link], [Link], [Link]).

The original BiB cohort are now adolescents, and since 2022 the BiB Age of Wonder (AoW) study has been collecting health and wellbeing data from up to 20,000 adolescents (including the BiB cohort and their peers) in mainstream education settings [41, 42, 43]. All aspects of the study have been co‐produced with groups of young people, teachers, parents, and community members. This has included co‐production groups' direct involvement in setting research priorities, co‐designing questionnaires, and presenting AoW findings at science festivals and in written publications (these activities are described in greater detail elsewhere [33, 42, 43]). Previous work has indicated a positive impact of co‐production both on co‐production group members engaging in AoW co‐production [33] and the project itself, with co‐production supporting alignment between AoW study delivery and the priorities of schools and young people [43]. However, no formal evaluation of AoW co‐production has been conducted through the first 3 years of the study. Given the need for co‐designed evaluations [28] and transparent reporting of co‐production practices [16, 26, 27], the lack of long‐term evaluations in the literature [27], and the limited reporting on co‐production with young people generally [16, 34], evaluating AoW co‐production represents a valuable opportunity to contribute to the evidence base.

### 1.2 Aim

This study aimed to evaluate co‐production in the BiB Age of Wonder study, using a mixed‐methods approach to assess how well the principles and values of co‐production have been embedded in AoW.

## 2 Methods

We followed the GRIPP2 checklist [44] for reporting public involvement in research (see Supporting Information: Appendices [Link], [Link], [Link], [Link]).

### 2.1 Study Design

A mixed‐methods design was employed, combining cross‐sectional survey data collection with focus group discussions. The study was co‐designed with young people from AoW co‐production groups during the summer of 2024. During initial co‐design sessions, the groups reflected on the core values and principles of the BiB co‐production strategy [37, 40] and developed questions they felt could assess how well these values and principles were embedded in AoW co‐production. Subsequent sessions focused on refining the survey tool to ensure the questions covered the nine principles but were not overly burdensome for prospective participants. The final survey tool included basic sociodemographic questions, 18 likert‐scale questions mapped onto the nine guiding principles, and three additional questions. These three additional questions were included based on group suggestions for open text questions, and previous research (both in AoW and elsewhere) highlighting the benefits co‐production can hold for those involved [33, 34, 45, 46]. Throughout the co‐design sessions, the groups emphasised the need for both closed and open‐ended questions; based on this advice, the evaluation was changed to a mixed‐methods approach and the open‐ended questions formed part of the focus group topic guide. The finalised survey tool and focus group topic guide are available in Supporting Information: Appendices [Link], [Link], [Link], [Link].

### 2.2 Participants

Participants were purposively recruited from existing AoW co‐production groups. This included the Bradford District Care Trust (BDCFT) Young Dynamos (school and university students aged 15–21), BiB young ambassadors (students aged 14–17 enroled in the Born in Bradford cohort), Young Leaders (young people 14–17 from Bradford community settings), the Healthy Minds Apprentices (young people aged 16–23 undergoing apprenticeships), BiB Parent Governors (parents of children enroled in the Born in Bradford cohort). Participants were eligible to take part so long as they had engaged in AoW co‐production activities since 2022, when the study started. All 112 eligible members across these groups were invited to take part.

### 2.3 Data Collection

An online survey was sent to all co‐production group members via email in November 2024. Survey data was collected using RedCap software. Four focus group discussions were held with co‐production group members in December 2024 and January 2025. Focus groups were conducted with three distinct groups to gather diverse perspectives and reflect the different groups involved in AoW co‐production; one group consisted of adults (parents, teachers, group facilitators), one group consisted of young people under 18 years of age, and the final two groups consisted of young adults aged 18–25. Focus group participants did not have any prior relationship with the focus group facilitators, in order to minimise observer effects [47]. Focus groups were audio‐recorded and transcribed verbatim.

### 2.4 Analysis

The survey contained 18 questions mapped evenly onto the nine principles of co‐production (i.e., two questions per principle) (see Table 2 for list of principles); internal consistency for each pair of questions was assessed using Spearman–Brown coefficients, with acceptability set at ≥ 0.7 [48]. Composite scores for each principle were created by averaging participants' responses for each relevant pair of questions. Quantitative analysis was undertaken using IBM SPSS Statistics software version 31 and involved two stages:

1. Descriptive statistics were conducted to assess how well participants thought the co‐production principles were embedded in AoW. Descriptive analysis were also conducted to summarise the demographic characteristics of the respondents and identify proportions of respondents reporting benefits of engaging in co‐production.
2. Friedman's tests were conducted to assess whether some principles were embedded more strongly than others. Wilcoxon signed‐rank tests with Bonferroni correction were used for post‐hoc pairwise comparisons between principles. Significance was accepted at *p* < 0.0014.

Focus group data was analysed using the seven stages of framework analysis, as outlined by Gale et al. [49]. These include the following stages: (1) transcription, (2) familiarisation with the transcript and/or recording, (3) coding, (4) developing a working analytical framework, (5) applying the analytical framework, (6) charting data into the framework matrix, and (7) interpreting the data. Transcripts were coded deductively using the nine co‐production principles as distinct ‘categories’ within the coding framework. Coding was done independently by two authors, to enhance the reliability and validity of the framework via cross‐verification of interpretations. Codes were assigned to one (or more) of the nine principles in the framework, along with any relevant researcher observations or notes. Sentiment analysis was also conducted, with codes categorised as positive, negative or neutral. Once both researchers had coded all transcripts (at least two iterations), initial interpretations were cross‐checked and synthesised (with input from additional members of the research team) to form one consolidated framework, showcasing how well (or not well) participants felt the nine co‐production principles were embedded in AoW, along with interpretation of associated barriers and enablers. This structured approach allowed for clear comparisons across focus groups and ensured that findings were linked to the co‐production values and principles. All qualitative analysis was conducted using NVivo version 14.

## 3 Results

A total of 79 of the 112 eligible participants completed the online survey, while 17 participants took part in one of four online focus groups. The survey sample were diverse in terms of sex, ethnicity and age; over 32% of respondents were of Asian or Asian British ethnicity, 44% were female, while over 75% were under 18 (see Table 1).

**Table 1:** Breakdown of co‐production evaluation survey and focus group participants.

	Surveys *n* (%)	Focus groups *n* (%)
Co‐Production Group		
Young Leaders	43 (54.4)	0 (0.0)
Young Dynamos	17 (21.5)	6 (35.3)
Healthy Minds Apprentices	6 (7.6)	5 (29.4)
Parent Governors	5 (6.3)	4 (23.5)
Young Ambassadors	5 (6.3)	2 (11.8)
Other^a^	3 (3.8)	0 (0.0)
Sex		
Male	44 (56.0)	5 (29.4)
Female	35 (44.0)	12 (70.6)
Age		
11–15 years	43 (54.4)	1 (5.9)
16–18 years	23 (29.1)	2 (11.8)
> 18 years	13 (16.5)	14 (82.3)
Ethnicity		
Asian or Asian British	26 (32.9)	9 (52.9)
Black or Black British	23 (29.1)	1 (5.9)
Mixed or Multiple ethnic groups	18 (22.8)	0 (0.0)
White	9 (11.4)	7 (41.2)
Other ethnic groups	3 (3.8)	0 (0.0)
Total	79 (100)	17 (100)

a Other category represents teachers/parents who have taken part in co‐production but aren't regular members of the aforementioned groups.

## 4 Embedding the Co‐Production Values and Principles in AoW Co‐Production

In an attempt to integrate the qualitative and quantitative perspectives, findings are described broadly under three inter‐related themes (equality, agency, reciprocity), and constituent sub‐themes (the nine guiding principles) corresponding to the three core values and principles of the co‐production strategy. It is worth noting that the principles and values should and are not considered in silos; they are inter‐related and participants' reflections can touch on one or more principles and values.

The following sections outline medians and means for each composite principle score, indicating to what extent participants felt the co‐production principles were embedded in AoW. Lower scores indicate stronger agreement that each principle has been embedded in AoW. Participants' reflections on AoW co‐production provide additional context, with supporting quotations. A participant key was used to protect participants' anonymity, listing the category (e.g., YA = young adult, YP = young person, PF = parent or facilitator) and participant number.

### 4.1 Equality

Whilst survey findings were positive for all equality‐related composite scores, the highest value across all principles was found for sharing power. Pairwise comparisons with a Bonferroni correction indicated a significant difference between sharing power and all other principles, except going to communities and not expecting them to come to you (see Supporting Information: Appendices [Link], [Link], [Link], [Link]). Together, these findings suggest that participants mostly agreed or strongly agreed that equality has been embedded in AoW, but highlighted sharing power as an area of improvement (see Table 2).

**Table 2:** Perceived embedding of equality‐related principles in AoW (1 = totally embedded, 5 = not at all embedded).

Co‐production value	Co‐production principle	*N*	Mean (SD)	Median (IQR)
Equality	Principle 1. Sharing Power	79	2.2 (0.9)	2.0 (1.5–2.5)
	Principle 3. Respecting and valuing knowledge and lived experience	79	1.6 (0.6)	1.5 (1.0–2.0)
	Principle 7. Appropriate terminology and information	79	1.7 (0.6)	2.0 (1.0–2.0)
Agency	Principle 2. Including all perspectives	79	1.6 (0.6)	1.5 (1.0–2.0)
	Principle 5. Going to communities and not expecting them to come to you	79	1.8 (0.6)	2.0 (1.0–2.0)
	Principle 6. Working flexibility	79	1.7 (0.7)	1.5 (1.0–2.0)
Reciprocity	Principle 4. Reciprocal benefits for all	79	1.8 (0.8)	2.0 (1.0–2.0)
	Principle 8. Building and maintaining long term relationships	79	1.7 (0.6)	1.5 (1.0–2.0)
	Principle 9. Adequate resources	79	1.8 (0.6)	1.5 (1.0–2.0)

Abbreviations: AoW, Age of Wonder; IQR, interquartile range; SD, standard deviation.

Equality was frequently referenced in the focus groups, and participants outlined mechanisms of embedding equality within sessions but also more broadly when co‐producing a research project. Participants spoke of how during sessions *“there's definitely a culture of respect”* and noted how there was “*no right or wrong answer”* **(Respecting and valuing knowledge and lived experience)**. Descriptions of sessions included:

“It's very informal, very relaxed, but at the same time you get stuff done, your voice is listened to, and you can make contributions that like nobody's gonna judge you for”

‐ *YP2*

“it's actually felt genuine, like when I've asked questions and it's taken onboard. I can see and I can feel that it's taken on board…I feel like I am being listened to which makes me feel like more empowered to speak as well”.

‐ *YA6*

AoW co‐production sessions were described by YA1 as being “more conversational” compared to other co‐production groups, while a YA4 described AoW co‐production as a “*very open conversation where you're allowed to be inquisitive without feeling like you're silly.”* An important part of the conversational approach described by participants was the omission of jargon **(Appropriate terminology and information)**, with both young people and parents/facilitators praising the use of inclusive language in AoW co‐production sessions and BiB more generally:

“We're always treated equally whilst also being spoken to in inclusive language that we can understand as young people”

‐ *YA7*

“I really admire the fact that Born in Bradford go out of their way to make sure that it is suitable and appropriate for young people”

‐ *PF5*

Participants also spoke positively about power being shared **(Sharing power)**, for example, YP2 spoke of how during sessions “*teenagers do the talking. We're the ones with the power instead of the co‐production leaders”. Speaking of co‐production more broadly, YA1 commented “we're all on the same page, we're all heading towards the same goal even though we are apprentices and these are researchers, but we don't feel inferior to them”*. However, YA7 believed that it would be helpful if they had the opportunity to *“have more suggestions on certain research rather than suggesting on the research projects that have already been decided”*, while PF5 commented how co‐production *“from start to finish and really seeing it through and getting the full experience, could be done a little bit better to make it feel more of a partnership.”*

Similarly, participants reported different perceptions of how their voice had an impact on the AoW study; for example YP2 reported how *“you can see what you've accomplished and how you've helped and that makes you come back for more, so you can help out as much as you can because you understand how beneficial your input is.”* However, some young adults had alternative perspectives, reporting that they would appreciate seeing more results that come from their input:

“Maybe by showing us the way that our voices have been put into action, because I feel like we give advice and sometimes we don't see the end result.”

‐ *YA4*

“Yeah, like highlighting changes that have been created as a result of co‐production. Like a you said, we did format. I think that would really push that young people are changing everything in BiB.”

‐ *YA6*

### 4.2 Agency

Survey findings suggest agency was well embedded in AoW with participants generally agreeing or strongly agreeing that constituent principles were present in AoW; however relatively high scores were found for going to communities and not expecting them to come to you, suggesting that this is a second area of potential improvement (see Table 2).

Focus group participants reported flexible approaches to co‐production **(Working flexibility)** both during and in between sessions through holding hybrid meetings, altering session timings, and taking a flexible approach to running sessions. Young adults mentioned having “*time to speak and work at our pace”* (YA1) and YA7 praised how *“meetings are always made so they fit around our lifestyles…and they make sure that we have time to get there and it's at times convenient for us even though they're giving up their evenings”*. Participants also mentioned working practices changing over the years; a notable example was provided by young adults who've transitioned from school to university:

“…with a lot of the [young people] now leaving and going off to university…you can see that AoW puts such an effort in making sure that it's a virtual and an in‐person meeting for the people that are still in Bradford and can join those meetings.”

‐ *YA4*

Some participants spoke about AoW co‐production including diverse voices **(Including all perspectives)**, as one group facilitator described:

“we have kids from different walks of life that might experience certain things that others might not experience, and I think it's important to gauge the scope of different young people”.

‐ *PF5*

However, this differed somewhat with accounts made by young people, who generally noted that AoW could do more to extend its reach, particularly in schools and communities **(Going to communities and not expecting them to come to you)**.

“I feel like it needs to be pushed in schools because I feel like nobody really knows about it and it's like a big, fantastic thing that is literally benefitting us…and it kind of helps all of us in a way.”

‐ *YP1*

Moreover, young people highlighted this engagement as an opportunity for young people already involved in AoW and/or AoW co‐production work:

“maybe instead of having adults involved in Age of Wonder go talk about it to schools, maybe have each participant who's involved go speak to their respective school that they attend, like tell them about Age of Wonder, that way they can promote Age of Wonder in a way that feels natural to them, they're promoting it to their peers who'll understand it more, and it will be a good experience for them in terms of public speaking and presentations.”

‐*YP2*

Some young adults highlighted a need to engage more young people in communities, to broaden the voices involved in co‐production activities.

“sometimes co‐production isn't really available for every young person in Bradford. It's available to some select groups…and maybe other young people might have other ideas and other perspectives and opinions that we can't really express. So, it might be important to include their voices as well, so like potentially going to youth centres and, you know, getting opinions from those young people there because they might not always engage with BiB in the same way that we do. So, I think that might be an area for improvement.”

‐ *YA6*

### 4.3 Reciprocity

Reciprocity was well embedded in AoW, with survey participants consistently agreeing or strongly agreeing that each constituent principle was present in AoW (see Table 2). Participants were also asked to list any benefits they had experienced from engaging in AoW co‐production. Inclusion of these questions was based on peer researcher input, and acknowledgement that co‐production can often generate implicit or ‘softer’ outcomes. Almost three quarters of participants reported gaining Increased Confidence (75%) and Research Knowledge (75%), while over two‐thirds reported developing skills (72%) and gaining use of their voice (68%). Finally, more than half (53%) reported making new friends through co‐production (see Figure 1).

**Figure 1:**
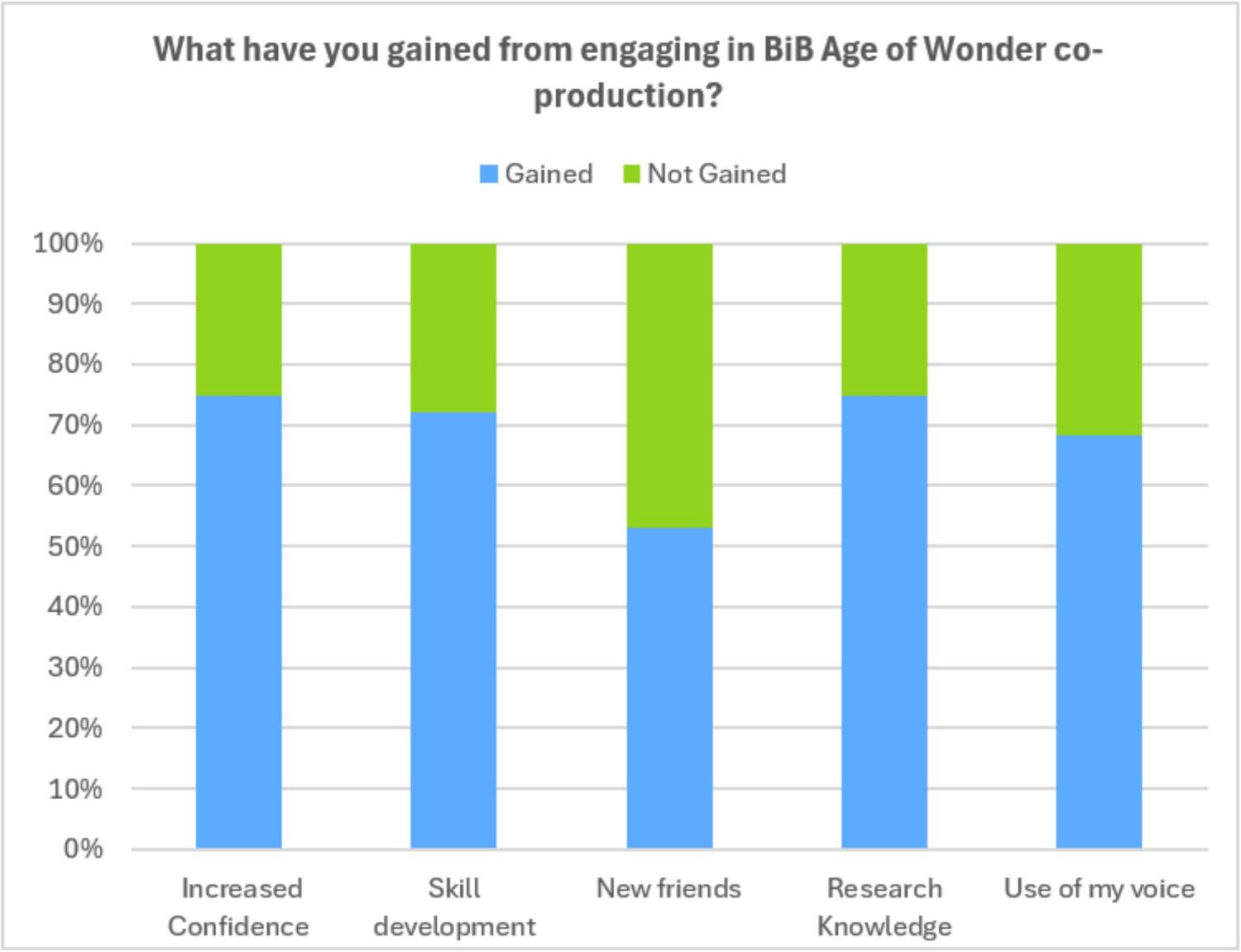
Percentages of participants who reported gaining something from co‐production engagement.

Focus group participants described their co‐production experience as a *“a win‐win process”* and “an equal exchange”. Notable examples of reciprocity **(Reciprocal benefits for all)** included young people experiencing personal development through attending regular sessions and bespoke opportunities. As one young person put it:

“it feels like a partnership, like you're both contributing to each other, they're helping you find more opportunities, develop your skills, have fun while doing it, make new friends, and you're helping them with the research, like giving your input, what you think is best, how they can improve”.

‐ *YP2*

One *y*oung adult (YA4) mentioned that co‐production had *“helped [them] grow so much as a person”, while YA6* described co‐production as a *“beginner way into research that allows you to understand concepts that would take you more time to study independently.”* For young adults, support gaining work experience was cited as an important benefit of co‐production:

“we're always offered opportunities, like if anyone's interested in this line of research for example, get in touch and we could sort something out, like work experience, which is so valuable”.

‐ *YA3*

While the co‐production experience was reported as largely positive and reciprocal, some participants expressed a desire to see greater reciprocity through alignment with young people's interests and more hands‐on experience:

“at the beginning of the year or when someone joins they could send out a survey about what people are interested in, like what they're studying, what are they interested in potentially studying, and then they can look at that and so if they get any opportunities they could post it”

‐ *YA3*

“I would love to see some lived experience research roles…seeing if young people can actually create research and go down that pathway to really allow the empowerment of young people, to get them doing what they need to do.”

‐ *YA6*

Participants also talked about AoW's efforts to foster relationships with the co‐production groups **(Building and maintaining long term relationships)**. An important enabler of this was the atmosphere within co‐production sessions, as one young person described:

“I think it's quite good the way they do it, like it's very relaxed, friendly atmosphere and the different types of research tasks that they do for children and teenagers are very relaxed, very sort of informal in a way”

‐ *YP2*

Similar sentiments were echoed by a young adult: *“there's a Christmas do so I'm looking forward to going back and I guess that reflects that, you know, I wouldn't really want to leave and they're definitely interested in people staying for the long‐term.”* ‐YA3

Group facilitators also spoke about enduring relationships created through the co‐production process:

“They have offered placements to my apprentices and I currently have one apprentice helping out there even though she's no longer part of our apprenticeship. She's moved on to university and she's still with AoW helping out when she can, which I think is great, keeping the relationships going.”

‐ *PF5*

Moreover, participants also highlighted the value of research teams supporting co‐production groups beyond the scope of the research project:

“I think BiB does a really good job. Before I even got involved in the co‐production aspect of it I actually had reached out to [AoW] to see if there were any opportunities when it came to placements or apprenticeships, anything like that and [AoW] really helped me to get into the role I am in now. So, I'm really grateful for that”

‐ *YA6*

“AoW's always really approachable, supportive, you know, they've even helped out with our group when we might be doing things that are not related to Age of Wonder”

‐ *PF3*

An important factor in enabling reciprocity and co‐production more generally was adequate resourcing **(Adequate resources)**. Generally, participants reported that AoW co‐production was well‐resourced, with YP2 saying, *“we have everything we need, we have all the right tools, pens, paper, everything that we need.”* However, one parent/facilitator noted that the time available for co‐production can be limited, particularly when considering young people's schedules:

“the young people we get usually have lots and lots of other activities that they're involved with as well, on top of their schoolwork. So that's our restriction really, is the world of young people.”

‐ *PF4*

## 5 Discussion

Co‐production is increasingly recognised as a dynamic, relational process where parties work iteratively and learn by doing [10, 50, 51]. As Langley et al. [50] state “co‐production research is not a technique you apply rightly or wrongly, but a journey of learning, and it is not a journey you make alone.” The present evaluation was conducted in the same vein. Researchers and co‐production group members co‐designed the study, and examined how well co‐production values and principles have been embedded in AoW.

Findings were generally positive and suggest that many, if not all of the BiB co‐production values and principles have been meaningfully embedded in AoW. Participants strongly agreed that Principles 6 (working flexibly), 7 (using appropriate language), and 9 (ensuring adequate resources) were present in AoW co‐production. Principles were reflected in relatively straight‐forward actions such as the use of inclusive and conversational language, working to young participants' schedules, and providing ample refreshments and session materials. Similar actions have been noted elsewhere [19, 20], illustrating simple steps for operationalising these co‐production principles.

Principles including valuing lived experience, ensuring mutual benefit, and building long‐term relationships were also present in AoW co‐production. However, when discussing these principles, participants highlighted a mixture of practical and more interpersonal enablers, ranging from offering research placements and opportunities, to creating open and non‐judgemental atmospheres, and ensuring participants felt listened to. Participants' reported benefits of co‐production included a similar mixture of practical benefits (increased research knowledge, skill development) and holistic benefits (increased confidence, making new friends, gaining use of their voice). These findings align with previous research emphasising the tacit, embodied nature of co‐production, the importance of simple gestures (e.g., knowing names, offering refreshments, thanking people for their time), and the need for researchers to view relationship building and reciprocity as central to effectively engaging co‐production groups [10, 15, 25, 26]. Interestingly, some participants valued activities that were actually separate to AoW research or co‐production (e.g., AoW researchers helping a young person gain work experience with another organisation, AoW researchers helping young people with their projects, holding a Christmas party). These examples further emphasise the importance of relationship building when working with young people, and echo previous evaluations highlighting how young people who may sometimes be mistrusting of researchers due to anticipated power dynamics and/or beliefs that their input will be ignored [16, 52].

Sharing power and going to communities emerged as areas of improvement. Participants called for earlier involvement in deciding research questions and shaping projects from the very beginning of the research process. They also expressed frustration at inconsistent or unclear feedback about how their input was applied. Suggestions for improvement included “you said, we did” feedback mechanisms and the creation of paid lived experience roles within the research team. Participants also advocated for greater engagement in schools and communities, both to increase inclusion and to enable upskilling via peer‐led engagement. Similar reflections have been reported in previous evaluations of youth involvement, including a lack of public involvement early in research design (especially young people) [16], the importance of regular feedback on young people's impact on projects [16], and tensions that can arise between diverse engagement and the number of people that can be viably included within constraints of time, budgets and staff capacity [53]. This suggests that these challenges extend beyond AoW to co‐production more generally, and further emphasise how putting co‐production principles into practice is not always straightforward or predictable, and places varying demands on research teams and organisations [10, 26, 54]. That said, participants' recommendations for simplified feedback loops and peer‐led approaches provide promising ways forward and will be integrated into future AoW co‐production practice.

### 5.1 Recommendations for Researchers and Practitioners

Findings from this paper provide some useful recommendations for researchers, ppie group facilitators, or anyone looking to engage young people in co‐production. Firstly, the overall positive sentiments shared by participants give further support to principle‐based approaches to co‐production, and as such following the core values and guiding principles of the BiB co‐production strategy is recommended as a useful starting point for researchers and/or practitioners looking to engage in co‐production activities, regardless of prior PPIE and/or co‐production experience. More specific recommendations from this evaluation include (1) building in ample budget and time to build relationships with groups and foster a conversational tone in sessions, (2) embracing operational flexibility to allow studies to morph as co‐production takes effect, (3) investing where possible in group members' upskilling and personal development even if this support extends beyond the immediate or obvious scope of the research project, and (4) coming to group consensus regarding how often groups meet, how long meetings are, the format of sessions, and how information is communicated. Finally, participants' suggestions for AoW improvements provide additional recommendations, namely providing regular and transparent feedback to groups, involving co‐production groups in studies during conceptualisation, and widening community engagement efforts as much as possible.

## 6 Strengths and Limitations

Co‐production is limited by a lack of transparent reporting on methods and challenges of enacting co‐production principles and values across unique contexts [16, 26, 27]. This study advances evidence by evaluating how values and principles of co‐production may or may not be embedded within a large complex adolescent health study spanning over several years. Other strengths of this work are the inclusion of participants in co‐designing the study, adoption of a mixed‐methods approach, and the inclusion of a diverse group of participants, particularly young people. Another strength of this work is its theoretical footing in the BiB co‐production strategy [37, 40], which itself underwent rigorous co‐design with community members. That said, the study does have some limitations. For example, whilst steps were taken to mitigate against Hawthorne/observer effects [47] this remains a limitation. Secondly, while the Spearman‐Brown coefficients indicated moderate internal consistency for most composite scores, some values fell below commonly accepted thresholds for reliability, suggesting that certain survey questions require further refinement to improve their measurement consistency. All participants were members of co‐production groups, therefore the insights presented in this paper only reflect those specific perspectives. For instance, co‐production groups may have limited awareness of budgetary constraints, funding timelines, or the influence of organisational ethos on the implementation and support of co‐production. Finally, it is worth noting that this evaluation explored how well principles and values were embedded in AoW, rather than how well co‐production did or did not improve AoW. Thus, these remain gaps in the co‐production evaluation evidence base.

## 7 Conclusion

Many, if not all of the BiB co‐production principles have been embedded in Age of Wonder (AoW) co‐production. Translating these principles into practice requires both practical actions (e.g., having session resources, flexible timelines) and sustained relational investment (e.g. using inclusive language, fostering conversational and informal atmospheres, supporting young people's development), particularly when working with young people. Despite these successes, challenges remain regarding equitable shaping of research agendas, improving feedback loops, and reaching wider communities and schools. Participants' calls for earlier involvement in study development, consistent clear feedback, and paid peer research roles offer clear pathways for progress. Clear structures, ongoing resourcing, and deep community engagement and partnering will be vital to these efforts. More broadly, future co‐production evaluations may benefit from integrating perspectives from researchers, peer researchers, members of the public and organisational decision‐makers, to better understand how co‐production is enacted and limited in practice.

## Author Contributions

**David Ryan:** conceptualisation, methodology, data curation, supervision, investigation, writing – original draft, writing – review and editing, visualisation, formal analysis, project administration, software, validation. **Adam Whitehead:** investigation, writing – original draft, writing – review and editing, formal analysis. **Amelia Hudak:** investigation, writing – original draft, writing – review and editing, formal analysis. **Edie Lee:** investigation, writing – original draft, writing – review and editing, formal analysis, data curation. **Isobel Dowling:** formal analysis, writing – review and editing, writing – original draft, investigation. **Jennie Ellul:** investigation, writing – original draft, writing – review and editing, formal analysis. **Jennifer Ingram:** investigation, writing – original draft, writing – review and editing, formal analysis. **Katy A. Shire:** conceptualisation, writing – review and editing, methodology, resources, supervision, project administration. **Shahid Islam:** conceptualisation, writing – review and editing. **John Wright:** conceptualisation, funding acquisition, writing – review and editing, supervision, resources. **Rosemary R. C. McEachan:** conceptualisation, funding acquisition, writing – review and editing, supervision, resources.

## Ethics Statement

The BiB Age of Wonder project has received ethical approval from the Bradford Leeds Research Ethics Committee [Ref [21]/YH/0261, date: 22.12.21]. All participants have provided written consent to be involved in the study; all authors have consented to being named and identified in this publication.

## Consent

All authors have consented for this paper being published.

## Conflicts of Interest

The authors declare no conflicts of interest.

## Supporting information


**Appendix 1:** GRIPP2 Checklist.

**Appendix 2:** Survey Questions & FG topic Guide.

**Appendix 3:** Internal Consistency Statistics.

**Appendix 4:** Matrix of Wilcoxon Signed‐Ranks Test Results.

## Data Availability

The data that support the findings of this study are available from the corresponding author upon reasonable request. The data sets used and/or analysed during the current study are available from the corresponding author on reasonable request.
