# Supplementary material for: Evaluation of Co‐Production in Adolescent Health Research: A Mixed Methods Study From Born in Bradford
Source: Health Expect. 2026 Oct 15;29(5):e70876. doi: 10.1111/hex.70876 (PMC13670425; doi:10.1111/hex.70876)
Supplement: Supplementary file 1 [file HEX-29-e70876-s001.docx]

**1. Populated GRIPP2 checklist**^44^ **for reporting public involvement in research**

| **Section and topic** | **Item** | **Reported on page No** |
| --- | --- | --- |
| 1: Aim | Report the aim of PPI* in the study | 4 |
| 2: Methods | Provide a clear description of the methods used for PPI in the study | 4-5 |
| 3: Study results | Outcomes—Report the results of PPI in the study, including both positive and negative outcomes | 4-5; 6-12 |
| 4: Discussion and conclusions | Outcomes—Comment on the extent to which PPI influenced the study overall. Describe positive and negative effects | 13-14 |
| 5: Reflections/critical perspective | Comment critically on the study, reflecting on the things that went well and those that did not, so others can learn from this experience | 14-15 |
