# Supplementary material for: Evaluation of Co‐Production in Adolescent Health Research: A Mixed Methods Study From Born in Bradford
Source: Health Expect. 2026 Oct 15;29(5):e70876. doi: 10.1111/hex.70876 (PMC13670425; doi:10.1111/hex.70876)
Supplement: Supplementary file 2 [file HEX-29-e70876-s002.docx]

**2. Co-designed Survey Questions and Focus Group Topic Guide**

Survey Questions:

| **No.** | **Question (original)** | **Answer categories** |
| --- | --- | --- |
| 1a | What age are you? | ( ) in years |
| 1b | * if answered ≥ 18 to question 1a  Please provide an email address we can contact you with: | Open text |
| 1c | * if answered < 18 to question 1a  Please provide your parent/guardian’s email address we can contact you with: | Open text |
| 1d | What is your ethnicity?  **Ethnicity is the racial and/or cultural background you associate with (e.g. White-English, Asian-Pakistani).**  *List all options | A) **White**  - English, Welsh, Scottish, Northern Irish or British - Irish - Gypsy or Irish Traveller - Roma - Polish - Slovakian - Romanian - Czech - Any other White background, write in  B) **Mixed or Multiple ethnic groups**  - White and Black Caribbean - White and Black African - White and Asian - Any other Mixed or Multiple background, write in  C) **Asian or Asian British**  - Indian - Pakistani - Bangladeshi - Chinese - Any other Asian background, write in  D) **Black, Black British, Caribbean or African**  - Caribbean - African background, write in below - Any other Black, Black British or Caribbean background, write in  E) **Arab**  - African Arab - Middle-eastern Arab   **F) Other Ethnic group**  *Please write in |
| 1e | The next questions are about sex and gender identity. In order for our research to be compared with other studies we have used standard measures similar to the National Census. This information is used to monitor equality between groups of people of different genders and sexes. Equality monitoring helps make sure that everyone is treated fairly and helps identify what services are needed.  You can choose not to answer any of the questions. If you feel that there is not an answer that applies to you, there will be a space for you to enter your own response.  What is your sex? (the biological sex on your birth certificate)  *A question about gender identity will follow in the survey.* | - female - male - do not wish to answer - wish to enter own response*   *space to self-describe |
| 1f | **Gender identity**  What is your gender? (how you would describe yourself) | - Female - Male - Non-binary - wish to enter own response*   *space to self-describe   - do not wish to answer |
|  | EVALUATION QUESTIONS |  |
| 2a | How long have you been involved in co-producing BiB Age of Wonder? | - __ in months |
| 2b | What BiB Age of Wonder co-production group are you a member of? | - Young Dynamos - Parent Governors - Young Leaders - Young Ambassadors - Healthy Minds Apprentices - Other*   *(please describe) |
| 2c | **How much do you agree or disagree with the statements?**  I have power to change the Age of Wonder project by making suggestions in co-production sessions  I have the same power as the Age of Wonder researchers when it comes to co-producing the Age of Wonder project (e.g. reviewing a consent form, changing a questionnaire, making decisions on some part of the project)  Age of Wonder co-production does a good job of bringing together diverse perspectives and skills (i.e. skills and perspectives from people of different ages, ethnicities, areas of Bradford district etc.)  During Age of Wonder co-production sessions, different perspectives and skills are embraced and supported  In Age of Wonder, the knowledge I have gained from my lived experience is valued and respected just as much as the knowledge researchers gain from studying/working in research  I can share my honest experience and opinions during co-production sessions and know that they will be respected and valued.  The Age of Wonder research team and I both benefit from being involved in Age of Wonder co-production activities  The Age of Wonder research team and I benefit the same amount from being involved in Age of Wonder co-production activities  I feel like the Age of Wonder team make enough effort to reach out to me personally.  I feel like the Age of Wonder team make enough effort to reach out to schools/communities.  I think Age of Wonder co-production sessions cater to different working styles and preferences (e.g. length of session, learning style, communication style, online or offline sessions)  The Age of Wonder team are flexible and open to change how they work to suit group members’ needs/preferences  I understand the information shared in Age of Wonder co-production sessions  The words used and information provided in Age of Wonder co-production sessions make it easy for me to give my input  The Age of Wonder team have gained my trust  The Age of Wonder team have built long-term relationships with me and my co-production group  I am given the right amount of time and/or support during and after co-production sessions to effectively co-produce the BiB Age of Wonder project  The financial support for Age of Wonder co-production is appropriate and fair (e.g. compensating groups for their time, having materials for sessions) | - Strongly agree - Agree - Neither agree nor disagree - Disagree - Strongly disagree |
| 2d | Please indicate which of these you have gained from engaging in Age of Wonder co-production (select all that apply) | - Increased Confidence - Skill development - New friends - Research Knowledge - Use of my voice - Nothing - Other*   * please describe |
| 2e | Please use this space to add any relevant context or explanation for your answers to the previous questions | - Open text |
| 2f | Please use this space to mention anything you feel is important regarding your experience of BiB Age of Wonder co-production: | Open text |

**Focus Group Topic Guide:**

**INTRO**

1. What does the term co-production mean to you?
2. How would you describe the Age of Wonder co-production process? [prompts: activities, pieces of research worked on, session format, session atmosphere]
3. How would you describe your involvement to this point with co-producing Age of Wonder? e.g. contributing to Age of Wonder, being involved in Age of Wonder?

**GENERAL REFLECTIONS**

4. In general, how do you feel about the Age of Wonder co-production work? How would you rate it? [prompt: can you write down your overall rating for Age of Wonder co-production from 1-10, 10 being perfect and 1 being terrible)
5. Can you explain your rating? What things affected the rating you assigned to Age of Wonder co-production?
6. What things about the Age of Wonder co-production process do you think have gone well? What have you enjoyed about the process?
7. What things about the Age of Wonder co-production process have gone less well? What things about the Age of Wonder co-production process do you think could be changed or improved?

**QUESTIONS ON PRINCIPLES OF CO-PRODUCTION**

8. How much effort do you think the Age of Wonder co-production team make to identify your strengths, interests, skills to support your involvement? Are there ways this could be better?
9. How much do you feel power is shared during Age of Wonder co-production process? Can you provide any examples of situations where power has/has not been shared appropriately? What impact did this have on you and/or the co-production process?
10. Do you feel you have been valued and respected in the AoW co-production sessions? Any specific examples of this? [Probes: What impact does this have on you/ on the co-production session/process? How can researchers show that your input is valued and respected?]
11. How well are the Age of Wonder team ensuring that their interactions with co-production groups are built for the long-term?
12. How much do you feel like you/your co-production group and Age of Wonder are partners? Does it feel like a real collaboration/partnership? Please explain your answer.
13. Who benefits from engaging in co-production for Age of Wonder? To what extent do you feel like you have benefited from your involvement in AoW co-production?
14. Is Age of Wonder co-production resourced enough? i.e. is there enough money, time, staff devoted to doing co-production? Are there any resources (e.g. money, time, staff) that Age of Wonder need more of in order to improve the Age of Wonder co-production process?

**FINAL QUESTION**

15. Are there any other things regarding the Age of Wonder co-production work that you feel are important to highlight? Are there any things the Age of Wonder team need to improve on? Or any suggestions for how to make Age of Wonder co-production better?
