# Supplementary material for: Evaluation of Co‐Production in Adolescent Health Research: A Mixed Methods Study From Born in Bradford
Source: Health Expect. 2026 Oct 15;29(5):e70876. doi: 10.1111/hex.70876 (PMC13670425; doi:10.1111/hex.70876)
Supplement: Supplementary file 3 [file HEX-29-e70876-s003.docx]

**4. Matrix of Wilcoxon Signed-Ranks Test Results**

|  | **Principle**  **1** | **Principle 2** | **Principle 3** | **Principle 4** | **Principle 5** | **Principle 6** | **Principle 7** | **Principle 8** | **Principle 9** |
| --- | --- | --- | --- | --- | --- | --- | --- | --- | --- |
| **Principle 1** | 0.00 | **-3.885*** | **-4.438*** | **-3.516*** | -3.049 | **-3.599*** | **-3.669*** | **-4.033*** | **-3.234*** |
| **Principle 2** | **-3.885*** | 0.00 | -0.454 | -1.943 | -2.068 | -0.96 | -0.788 | -0.491 | -1.776 |
| **Principle 3** | **-4.438*** | -0.454 | 0.00 | -2.291 | -2.108 | -1.796 | -1.067 | -0.778 | -1.956 |
| **Principle 4** | **-3.516*** | -1.943 | -2.291 | 0.00 | -0.581 | -1.009 | -1.374 | -1.351 | -0.841 |
| **Principle 5** | -3.049 | -2.068 | -2.108 | -0.581 | 0.00 | -1.312 | -1.37 | -1.662 | -0.223 |
| **Principle 6** | **-3.599*** | -0.96 | -1.796 | -1.009 | -1.312 | 0.00 | -0.084 | -0.302 | -0.734 |
| **Principle 7** | **-3.669*** | -0.788 | -1.067 | -1.374 | -1.37 | -0.084 | 0.00 | -0.288 | -0.971 |
| **Principle 8** | **-4.033*** | -0.491 | -0.778 | -1.351 | -1.662 | -0.302 | -0.288 | 0.00 | -1.015 |
| **Principle 9** | **-3.234*** | -1.776 | -1.956 | -0.841 | -0.223 | -0.734 | -0.971 | -1.015 | 0.00 |

*Indicates statistically significant result at p < 0.0014
