# Supplementary material for: Evaluation of Co‐Production in Adolescent Health Research: A Mixed Methods Study From Born in Bradford
Source: Health Expect. 2026 Oct 15;29(5):e70876. doi: 10.1111/hex.70876 (PMC13670425; doi:10.1111/hex.70876)
Supplement: Supplementary file 4 [file HEX-29-e70876-s004.docx]

**3. Internal Consistency for Each Principle Composite Score**

| **Principle** | **Spearman-Brown Coefficient** |
| --- | --- |
| **Principle 1** | 0.869 |
| **Principle 2** | 0.724 |
| **Principle 3** | 0.839 |
| **Principle 4** | 0.684 |
| **Principle 5** | 0.544 |
| **Principle 6** | 0.737 |
| **Principle 7** | 0.764 |
| **Principle 8** | 0.771 |
| **Principle 9** | 0.639 |
